# Targeted Metabolomics Analysis of Individuals Carrying the ANGPTL8 R59W Variant

**DOI:** 10.3390/metabo13090972

**Published:** 2023-08-24

**Authors:** Mohamed Abu-Farha, Shibu Joseph, Anwar Mohammad, Arshad Channanath, Ibrahim Taher, Fahd Al-Mulla, Muhammad Mujammami, Thangavel Alphonse Thanaraj, Jehad Abubaker, Anas M. Abdel Rahman

**Affiliations:** 1 Department of Biochemistry and Molecular Biology, Dasman Diabetes Institute, Dasman 15462, Kuwait; mohamed.abufarha@dasmaninstitute.org (M.A.-F.); shibu.joseph@dasmaninstitute.org (S.J.); anwar.mohammad@dasmaninstitute.org (A.M.); 2 Department of Genetics and Bioinformatics, Dasman Diabetes Institute, Dasman 15462, Kuwait; arshad.channanath@dasmaninstitute.org (A.C.); fahd.almulla@dasmaninstitute.org (F.A.-M.); 3 Microbiology Unit, Department of Pathology, College of Medicine, Jouf University, Sakaka 72388, Saudi Arabia; itaher@ju.edu.sa; 4 Endocrinology and Diabetes Unit, Department of Medicine, College of Medicine, King Saud University, Riyadh 11421, Saudi Arabia; mhmujammami@ksu.edu.sa; 5 University Diabetes Center, King Saud University Medical City, King Saud University, Riyadh 11421, Saudi Arabia; 6 Metabolomics Section, Department of Clinical Genomics, Centre for Genome Medicine, King Faisal Specialist Hospital and Research Centre (KFSHRC), Riyadh 11211, Saudi Arabia; aabdelrahman46@kfshrc.edu.sa; 7 Department of Biochemistry and Molecular Medicine, College of Medicine, Alfaisal University, Riyadh 11533, Saudi Arabia; 8 Department of Chemistry, College of Science, Memorial University of Newfoundland, St. John’s, NL A1C 5S7, Canada

**Keywords:** ANGPTL8, R59W variant, rs2278426 SNP, metabolites, metabolome, inflammation, Arabs, Kuwait

## Abstract

ANGPTL8 is recognized as a regulator of lipid metabolism through its role in inhibiting lipoprotein lipase activity. ANGPTL8 gene variants, particularly rs2278426 leading to the R59W variant in the protein, have been associated with lipid traits in various ethnicities. We aimed to use metabolomics to understand the impact of the ANGPTL8 R59W variant on metabolites in humans. We used the Biocrates-p400 kit to quantify 408 plasma metabolites in 60 adult male Arab individuals from Kuwait and identify differences in metabolite levels between individuals carrying reference genotypes and those with carrier genotypes at ANGPTL8 rs2278426. Individuals with carrier genotypes (CT+TT) compared to those carrying the reference genotype (CC) showed statistically significant differences in the following metabolites: acylcarnitine (perturbs metabolic pathways), phosphatidylcholine (supports liver function and cholesterol levels), cholesteryl ester (brings chronic inflammatory response to lipoprotein depositions in arteries), α-aminoadipic acid (modulates glucose homeostasis), histamine (regulates glucose/lipid metabolism), sarcosine (links amino acid and lipid metabolism), diacylglycerol 42:1 (regulates homeostasis of cellular lipid stores), and lysophosphatidylcholine (regulates oxidative stress and inflammatory response). Functional aspects attributed to these metabolites indicate that the ANGPTL8 R59W variant influences the concentrations of lipid- and inflammation-related metabolites. This observation further highlights the role of ANGPTL8 in lipid metabolism.

## 1. Introduction

The menaces of type 2 diabetes (T2D) and obesity have reached alarming levels in almost all global populations [1,2]. These menaces are often associated with abnormalities in metabolism processes, resulting in high levels of triglycerides (TG) and free fatty acids. ANGPTL8 has been identified as a regulator of triglyceride metabolism through its inhibition of lipoprotein lipase activity. The expression of ANGPTL8, which is modulated by food intake, is abundant in adipose tissue and the liver. Zhang et al. have shown that ANGPTL8 gene expression is enhanced in mouse hepatocytes and matured adipocytes [3]. As high as a 35-fold increase in the expression is seen in adipocytes upon insulin therapy [4]. In our previous study using a large cohort of 1049 non-diabetic people and 556 people with T2D, we demonstrated that the ANGPTL8 level was nearly three times higher in people with T2D [5]. Furthermore, ANGPTL8 is positively associated with many factors such as age, body mass index (BMI), waist/hip ratio, fasting blood glucose (FBG), HbA1c, and homeostatic model assessment for insulin resistance (HOMA-IR) in non-diabetic people. The results highlight that an elevated level of ANGPTL8 in diabetes led to neither elevation in insulin production nor an alteration in glucose levels [5]. In addition to this, we have found that ANGPTL8, while correlating positively with C-peptide levels in healthy individuals, is not associated with them in T2D individuals [6]. A Japanese study on cohorts of 34 T1D and 30 T2D individuals demonstrated higher levels of ANGPTL8 in diabetic subjects than in non-diabetic subjects [7]. There are many other research studies that show that ANGPTL8 was increased in T2D individuals [7,8,9]. 

Previous genome-wide association studies have associated the *ANGPTL8* rs2278426_ c.175C.T_p.R59W variant with low-density cholesterol, high-density cholesterol, total cholesterol, and triglycerides in global populations, such as those of European ancestry [10,11,12], East Asian ancestry [13,14,15,16,17], and Hispanic ancestry [18], as well as in multi-ethnic cohorts of Europeans and East Asians [13,19]; of individuals with European ancestry, African ancestry individuals, and South Asian ancestry from the United Kingdom [20]; and of African American individuals, Hispanic individuals, East Asian individuals, Oceanian individuals, Native American individuals, and European individuals from the USA [21,22,23]. The variant has also been shown to be associated with the waist-to-hip ratio in individuals of European ancestry [24]. We recently observed that fasting blood glucose levels in Arab individuals with the CT carrier genotype at rs2278426 were higher compared to those with the reference CC genotype [25]. We further demonstrated that the level of circulating ANGPTL8 in people with metabolic syndrome is positively correlated with levels of hsCRP, implicating ANGPTL8 in metabolic and inflammatory pathways [26]. In recent work, we demonstrated, through genetic association tests, that the ANGPTL8 R59W variant is associated with increased circulatory levels of TNFα and IL7 and with increased activity of NF-κB p65; further, through in vitro assays and structural studies, we demonstrated that the variant could modulate the NF-κB signaling pathway and thereby regulate proinflammatory events [27]. Inflammation is a critical factor in the etiology of metabolic disorders, including obesity, type 2 diabetes, and atherosclerosis.

As mentioned above, while several global genome-wide association studies exist relating the ANGPTL8 R59W variant with lipid traits, only a couple of studies exist relating ANGPTL8 (and not the R59W particularly) with diabetes. Thus, it may be considered that the inference on the association of the particular variant with lipid metabolism has more impact and that the role of ANGPTL8 and the R59W variant in diabetes needs to be further clarified by more studies. Thus, the comprehensive characterization of the variant’s role in various metabolic pathways remains an active area of research.

Metabolite concentrations and profiles have a strong genetic predisposition [28,29]. Metabolites are reaction precursors, intermediates, and products of metabolism. Altered metabolite levels can cause clinical symptoms and diseases, and such metabolites may serve as therapeutic targets to prevent and treat these diseases. Identification of such metabolites, whose levels are altered, may indicate the pathways and/or cell types that are altered in the disease state. Given the published role of the ANGPTL8 R59W variant in metabolic traits and inflammation, it would be informative to identify the metabolites whose concentrations are altered by the ANGPTL8 R59W variant. In this study, we aimed to assess the impact of the variant on the levels of 408 plasma metabolites via metabolomics in adult Arab individuals from Kuwait. 

## 2. Materials and Methods

### 2.1. Participant Recruitment 

The Ethical Review Committee of Dasman Diabetes Institute reviewed and approved the study by adopting the guidelines of the Declaration of Helsinki and the US Federal Policy for the Protection of Human Subjects. 

The cohort used in the study is a subset of the one reported in our previous study [27]. A set of 60 individuals were randomly selected to represent a nearly equal number of reference genotypes and carrier genotypes at the ANGPTL8 rs2278426_p.R59W variant. The main cohort was recruited by way of a random sampling of native Arab adult individuals from the six governorates of Kuwait. Exclusion criteria included pregnant women and individuals with chronic disorders. Informed consent was obtained from every participant. 

### 2.2. Collection and Processing of Blood Samples

EDTA-treated tubes were used to store blood samples collected from participants who had fasted overnight. We extracted the DNA using the Gentra Puregene^®^ kit (Qiagen, Valencia, CA, USA) and the Quant-iT™ PicoGreen^®^ dsDNA Assay Kit (Life Technologies, Grand Island, NY, USA). A BioTek Epoch Microplate Spectrophotometer (Agilent Technologies, Santa Clara, CA, USA) was used to quantify the extracted DNA. Adherence to the optical density range of 1.8–2.1 was confirmed by measuring absorbance values at 260–280 nm. 

### 2.3. Targeted Genotyping of the ANGPTL8 Study Variant rs2278426 

The protocol used to genotype the rs2278426 SNP (R59W variant) in the cohort included the use of the TaqMan^®^ Genotyping Assay kit on the ABI 7500 Real-Time PCR System (Foster City, CA, USA). Details on the experimental conditions and procedures for targeted genotyping and Sanger sequencing to validate selected cases of genotypes (homozygous for minor allele and heterozygous) are as described in our previous publication [27]. 

### 2.4. Metabolomics

We used the Absolute IDQ-p400 HR kit (Biocrates Life Science AG, Innsbruck, Austria) to perform targeted metabolomics. The kit enabled us to examine quantitative data on 408 metabolites covering 11 different classes. The listing of these 11 classes and detailed descriptions of the experimental techniques (including Liquid Chromatography-High Resolution Mass Spectrometry, Flow Injection Analysis) and the quantification methods (MetIDQ StatPack module) are as elaborated in our previous publication [30]. 

### 2.5. Statistical Analysis

The MetaboAnalyst tool (McGill University, Canada) was used to analyze the metabolite data. Normalization of the data was carried out as described in [30]. Data on the levels of metabolites for each study subject along with the genotype at the study variant is provided in Table S1. Comparisons between study participants with or without the variant were performed using Fisher’s exact test (for categorical variables), Pearson’s Chi-squared test (for categorical variables, *n* > 20), the Wilcoxon rank-sum exact test (for median variables, *n* < 20), and the Wilcoxon rank-sum test (for median variables, *n* > 20). Differences were considered statistically significant when *p* values were <0.05.

## 3. Results

### 3.1. Clinical Characteristics of the Study Cohort and the Sub-Cohorts

The clinical characteristics of the subjects from the study cohort are summarized in Table 1. The cohort comprised 60 participants, all of whom were male individuals. The median (IQR) age of the participants was 42 (37, 52) years. The cohort comprised mostly overweight and obese participants with a median (IQR) BMI of 29.0 (26.6, 32.2) kg/m^2^ and a WC of 94 (90, 106) cm. Median HbA1c (5.5%), low-density lipoprotein (3.62 mmol/L), high-density lipoprotein (0.99 mmol/L), total cholesterol (5.47 mmol/L), and triglyceride levels (1.21 mmol/L) were normal or near optimal. Furthermore, 47% of the participants were obese, and 23% were pre-diabetic; there were no diabetic patients in the cohort. Sub-dividing the study cohort into two sub-cohorts based on the genotype (reference genotype, CC, or the carrier genotypes, CT+TT) at the study variant indicated that the two groups were matched for all the measured clinical traits (see Table 1).

The mean value of triglyceride (TG) levels in the participants with the R59W variant, at 1.61 mmol/L, is higher than that of the participants (1.13 mmol/L) with the wild-type genotype. Regarding the total cholesterol (TC), the mean value is high at 5.47 mmol/L irrespective of the genotype. High TG accompanied by high TC seen in individuals with the R59W variant is characteristic of hyperlipidemia (dyslipidemia). 

### 3.2. Differences in the Levels of Metabolites between Subjects with the Reference CC Genotype and Those with the Carrier (CT+TT) Genotypes

**Figure 1:**
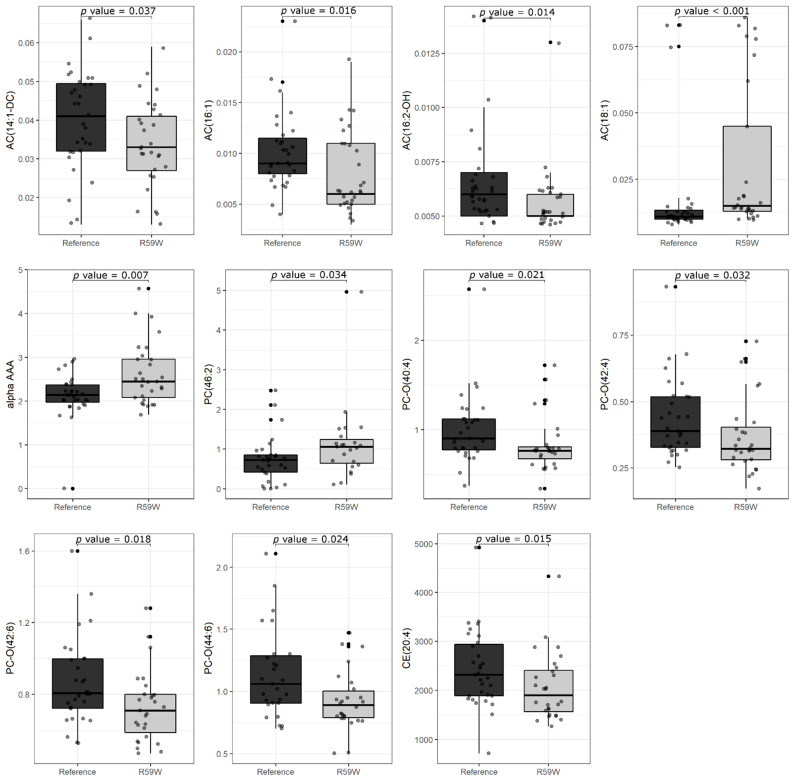
Boxplots for metabolite levels are significantly different between the two sub-cohorts of reference genotype CC and carrier genotype (CT+TT). The figure displays multiple boxplots representing the differences in metabolite levels between the CC genotype (as Reference) and CT+TT genotype (as R59W). The statistical significance of the difference between the metabolite levels of individuals with the reference CC genotype and those with the carrier genotypes is indicated by *p* values.

Of the 408 metabolites, the genotype-wide distributions of metabolite levels are significantly (*p* < 0.05) different in the case of 11 metabolites (Table 2 and Figure 1). These 11 metabolites fall into four categories. 

(i) Acylcarnitines (AC.18.1): The AC metabolites are fatty-acyl chains connected to carnitine molecules via an ester bond; these ester bonds are oxidized via fatty acid oxidation. It is known that perturbations in metabolic pathways elevate plasma and tissue levels of acylcarnitines [31]. Experiments with RAW 264.7 cells indicated that mixed D,L isomers of C12-/C14-carnitine activate the NF-κB-luciferase reporter gene, suggesting potential activation of proinflammatory pathways [32].

(ii) Phosphatidylcholine species (PC.40.4.): Phosphatidylcholine (PC) is a phospholipid attached to a choline particle. Phospholipids are composed of fatty acids, phosphorous, and glycerol. Apart from their traditional use in improving brain health, the PC species are implicated in liver function and in controlling cholesterol levels. Phosphatidylcholine is known to reduce the TNF-α-induced upregulation of pro-inflammatory cytokines in intestinal epithelial cells [33]. The cholesterol/PC ratio tunes processes such as biosynthesis and efflux of cholesterol, storage of cholesteryl esters in lipid droplets, and uptake of plasma lipoproteins [34].

(iii) Cholesteryl Ester (CE.20.4., also known as cholesteryl palmitic acid): Atherosclerosis, which is a chronic inflammatory response to lipoprotein depositions in artery walls, is characterized by accumulation of cholesterol esters in the arterial intima. CE may also accumulate in hereditary hypercholesterolemia. [35,36]. It is known that palmitic acid can stimulate the production of TNF-*α*, interleukin 6, and interleukin 1*β* in HaCaT keratinocytes and cell proliferation [37]. 

(iv) α-AAA (α- or 2-Aminoadipic acid): This metabolite is represented as an intermediate product in the α-Aminoadipic acid pathway of lysine metabolism. It has been identified as a novel biomarker for diabetes and it potentially modulates glucose homeostasis [38].

**Figure 2:**
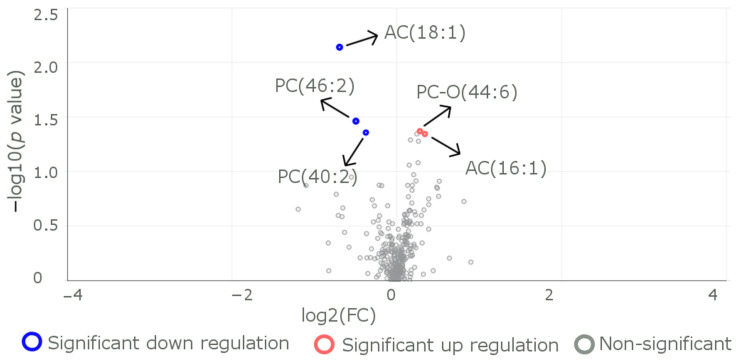
Metabolites that exhibit differences in levels between the individuals of reference genotype CC and individuals of heterozygous carrier genotype CT. A volcano plot represents log2(fold change) on the *x*-axis and −log10(*p* value) on the *y*-axis to visualize the differentially expressed metabolites between CC (*n* = 31) and CT (*n* = 26) genotypes. The plot indicates that five molecules showed significant differential expression; two of them were downregulated (represented by red dots), while three were upregulated (represented by blue dots) in the R59W variant wheS1n compared to the CC genotype. A fold change cutoff of 1.2 was applied to highlight changes in expression levels, while a *p* value threshold of less than 0.05 was used to define statistical significance.

We further divided (at the cost of losing statistical power) the cohort into three sub-cohorts of reference CC genotype, heterozygous carrier genotype CT, and homozygous carrier genotype TT at the study variant *ANGPTL8* rs2278426. Comparing metabolite levels between individuals carrying the CC genotype and individuals carrying the CT genotype revealed the PC-O (44:6) (belonging to the Phosphatidylcholine species) metabolite as having differential levels (Figure 2) in addition to the ones (AC (16:1), AC(18:1), PC(40:2), and PC(46:2)) identified above. 

(v) PC-O, 1-O-alkyl-2-acylglycerophosphocholine, belongs to the Phosphatidylcholine species. PC-O 44:6 has been suggested as a useful biomarker to detect and quantify visceral adiposity [39]. 

**Figure 3:**
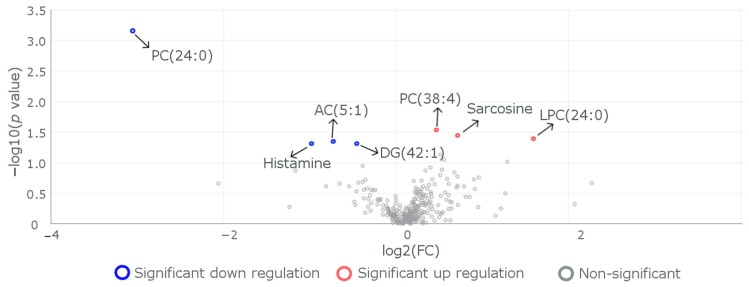
Metabolites that exhibit differences in levels between the individuals of reference genotype CC and individuals of heterozygous carrier genotype TT. A volcano plot represents log2(fold change) on the *x*-axis and −log10(*p* value) on the *y*-axis to visualize the differentially expressed metabolites between CC (*n* = 31) and TT (*n* = 3) genotypes. The plot indicates that seven molecules showed significant differential expression; three of them were downregulated (represented by red dots), while four were upregulated (represented by blue dots) in the R59W variant when compared to the CC genotype. A fold change cutoff of 1.2 was applied to highlight changes in expression levels, while a *p* value threshold of less than 0.05 was used to define statistical significance.

Comparing metabolite levels between individuals carrying the CC genotype and individuals carrying the TT genotype revealed the metabolites histamine, DG (42:1), sarcosine, and LPC (24:0) as having differential levels (Figure 3), in addition to the AC (5:1) and PC (38:4) that were identified when we compared the individuals carrying CC genotype with those carrying (CT+TT) genotypes. 

(vi) Histamine: Histamine belongs to the class of 2-arylethylamines. Histamine has a critical role in the regulation of energy intake and expenditure. Histamine signaling regulates the metabolism of glucose and lipids and promotes hyperlipidemia-induced NASH [40]. 

(vii) DG (42:1), diacylglycerol 42:1: A critical factor in the homeostasis of cellular lipid stores and membranes is the regulation of glycerolipid biosynthesis. Most common forms of insulin resistance, relating to obesity, T2D, lipodystrophy, and aging, can be explained by diacylglycerol mediation [41].

(viii). Sarcosine: Sarcosine belongs to the class of alpha amino acids. Sarcosine has been identified as a critical node that links amino acid and lipid metabolism in aged animals administered with sarcosine supplementation. Sarcosine, at the nexus of folate, Met, and glycine metabolism, acts as a biomarker for aging and metabolic phenotype in diet restriction [42].

(ix) LPC (24:0): Lysophosphatidylcholine (LPC) belongs to the class of glycerophospholipids. LPCs are products of phosphatidylcholine cleavage via the action of phospholipase A, and/or of fatty acid transfers to free cholesterol via lecithin–cholesterol acyltransferase [43].

## 4. Discussion

The role of ANGPTL8 in metabolizing lipids and glucose and in metabolic disorders and inflammation is widely recognized [7,8,9,27,44,45,46]. The rs2278426, while a missense (R59W) variant in *ANGPTL8*, is located in the intron in the *DOCK6* gene. The variant downregulates CTC-510F12.4 (a novel transcript) in whole blood, upregulates *DOCK6* in subcutaneous adipose tissue, and is associated with lipid traits and waist-to-hip ratio, as listed in the GWAS Catalog [47] and as enumerated in the introduction. A study of an Arab cohort found that the study variant and another variant, rs737337 from *ANGPTL8*, are associated with a lower risk of hypercholesterolemia and hyperglycemia [48]. Additionally, a study on Japanese cohorts, by way of demonstrating higher rates of T2D and impaired glucose tolerance in individuals with the R59W variant, highlighted the variant as a potential target for the prevention of T2D [49]. However, studies on the impact of this variant on these disorders through altering the levels of metabolites are lacking in the literature. Our study is the first to investigate the metabolite profile of the R59W variant and to interpret the role of metabolites, the levels of which are modulated by the R59W variant, in lipid metabolism and inflammation. 

In the present study, we identified the following metabolites—which seem to be often related to inflammatory pathways—with differential levels between individuals carrying the R59W variant and those not carrying the variant: acylcarnitines (perturbations in metabolic pathways), phosphatidylcholine (supporting liver function and cholesterol levels), cholesteryl the ester (chronic inflammatory response to deposition of lipoproteins in arteries), α-aminoadipic acid (modulator of glucose homeostasis), diacylglycerol (mediates insulin resistance associated with obesity and T2D, as well as lipodystrophy), histamine (histamine signaling can regulate glucose and lipid metabolism), sarcosine (a critical node linking amino acid and lipid metabolism), and lysophosphatidylcholine (a class of lipid biomolecule). Studies have shown that acylcarnitines activate proinflammatory signaling pathways through the activation of cyclooxygenase2 in RAW 264.7 cells [32]. The same group also revealed that these mixed isomers of C12 or C14 activate proinflammatory pathways through the NF-κB luciferase reported gene [32]. Our study shows that individuals carrying this R59W variant have increased levels of acylcarnitine (AC 18.1) as compared to the wild-type. Low levels of phosphatidylcholine were shown in patients with ulcerative colitis [50], while clinical studies revealed that phosphatidylcholine-rich preparations were beneficial for ulcerative colitis patients [50]. In vitro studies in human intestinal cells have shown that phosphatidylcholine blocks the TNF-α-induced activation of proinflammatory cytokines, representing a beneficial role in immunity [50]. In support of this, our studies have shown that individuals having the R59W variant have lower phosphatidylcholine levels as compared to the wild-type, thereby indicating that the protective role of phosphatidylcholine (as shown in Table 2 for P.C.O.40.4, P.C.O.42.4, P.C.O.42.6, P.C.O.44.6) is masked in individuals having carrier genotype. Few in vitro studies have shown the effects of inflammation and cholesterol metabolism; they studied the effect of LPS treatment in hepatocellular carcinoma HCC cells and found that the treatment induced an increase in intracellular cholesterol accumulation by the NF-κB pathway [51]. Interestingly, in our study, we found that there was a decrease in the cholesteryl ester for individuals with the heterozygous genotype as compared to the wild-type in circulation. Histamine is an inflammatory mediator that impacts the immune system as a proinflammatory factor. Histamine seems to have dualistic effects: (i) histamine, by way of activating pathways that promote the production of inflammatory mediators and cytokines, affects inflammation of the immune system, and (ii) the innate and adaptive immune responses are regulated by histamine [52]. Sarcosine, known to be part of the pathway of glycine, serine, and threonine metabolism, significantly associates with the levels of the inflammatory marker IL8 [53]. Intracellular accumulation of diacylglycerol, in muscle and liver, impairs insulin signaling by activating protein kinases C [54]. Lysophosphatidylcholinses are known as critical mediators of homeostasis involved in vascular inflammation [55].

Further, a genome-wide association study to identify genomic regions associated with metabolites in Hispanic individuals by Feofanova et al. [56] identified that the T allele at the study variant (rs2278426-T, leading to the R59W amino acid change in ANGPTL8) was associated with a decrease in (1-stearoyl-2-arachidonoyl-GPI (18:0/20:4)), and the reference allele at the variant was associated with an increase in (1-stearoyl-2-oleoyl-GPI (18:0/18:1). Both these metabolites belong to the superclass of glycerophospholipids (main class: glycerophosphoinositols; subclass: phosphatidylinositols). Glycerophosphoinositols are known to inhibit lipopolysaccharide-induced inflammatory and thrombotic responses [57]. Further, lysophosphatidylcholine (LPC), the other metabolite with differential levels depending on the R59W variant, is a member of the group of bioactive lipids that have been extensively studied for their role in inflammation and atherosclerosis [55].

In summary, the connection between the observed results on metabolite level alteration due to the R59W variant and the risk for disorders is described below. The mean value of triglyceride levels in the participants with the R59W variant, at 1.61 mmol/L, is higher than that of the participants (1.13 mmol/L) with the wild-type genotype. Regarding the total cholesterol, the mean value is high at 5.47 mmol/L irrespective of the genotype. High TG accompanied by high TC seen in individuals with the R59W variant is characteristic of hyperlipidemia (dyslipidemia). Our earlier work [27] demonstrated the association of the variant with increased circulatory levels of TNFα and IL7. The activity of NF-κB was elevated in individuals with the variant. It is known that NF-κB induces the expression of various pro-inflammatory genes and participates in inflammasome regulation. Inflammation is a critical factor in the etiology of metabolic disorders, including obesity, type 2 diabetes, and atherosclerosis [58]. The impact on the risk for disorders due to the observed alteration in metabolite levels in individuals with the R59W variant is presented in Table 3. The disorders for which the altered metabolite may act as a risk factor of biomarker include atherosclerosis, cardiometabolic disorders, diabetes, cellular senescence, and certain types of cancer.

The work reported in the presented study and in the literature reports discussed in this manuscript illustrates that the R59W variant can alter the levels of lipids (which can result in dyslipidemia), proinflammatory markers (that are critical factors in the etiology of metabolic disorders including obesity, type 2 diabetes, and atherosclerosis), and metabolites (that are risk factors and biomarkers for atherosclerosis, cardiometabolic disorders, diabetes, cellular senescence, and certain types of cancer). Thus, genotyping the R59W variant can be considered by clinicians evaluating patients with symptoms of the above-mentioned disorders. 

## 5. Conclusions

Our study demonstrates that the ANGPTL8 R59W variant alters the levels of metabolites that are implicated in lipid metabolism and inflammation processes. 

## Figures and Tables

**Table 1:** Clinical characteristics of the study cohorts.

Characteristics	All the Participants, *n* = 60 ^1^	Participants with the Variant R59W, *n* = 29 ^1^	Participants with the Reference Genotype, *n* = 31 ^1^	*p* Value ^2^
Genotype at the variant				<0.001
CC	31 (52%)	0 (0%)	31 (100%)	
CT	26 (43%)	26 (90%)	0 (0%)	
TT	3 (5.0%)	3 (10%)	0 (0%)	
Age: years	42 (37, 52)	42 (38, 53)	43 (37, 51)	0.8
Sex: male	60 (100%)	29 (100%)	31 (100%)	
Height: cm	170 (166, 175)	171 (169, 176)	169 (164, 175)	0.2
Weight: Kg	86 (78, 96)	88 (80, 99)	82 (77, 95)	0.14
BMI: Kg/m^2^	29.0 (26.6, 32.2)	30.8 (26.5, 32.5)	28.0 (26.7, 31.3)	0.3
WC (cm)	94 (90, 106)	94 (92, 103)	98 (90, 107)	0.8
Hip (cm)	103 (101, 110)	103 (102, 110)	104 (101, 109)	>0.9
SBP	38 (0, 136)	103 (0, 135)	0 (0, 136)	0.5
DBP	26 (0, 87)	59 (0, 88)	0 (0, 82)	0.6
FBG: mg/dL	90.81 (82.88, 98.90)	89.19 (82.88, 99.64)	92.61 (83.24, 97.30)	0.9
FBG: mmol/L	5.04 (4.60, 5.47)	4.95 (4.60, 5.53)	5.14 (4.62, 5.40)	0.9
HbA1C: %	5.50 (5.30, 5.70)	5.56 (5.38, 5.62)	5.40 (5.00, 5.77)	0.6
TC: mmol/L	5.47 (4.65, 5.70)	5.47 (4.75, 5.70)	5.45 (4.21, 5.83)	0.9
TG: mmol/L	1.21 (0.97, 2.33)	1.61 (0.97, 2.50)	1.13 (0.99, 2.04)	0.6
HDL: mmol/L	0.99 (0.83, 1.19)	1.04 (0.84, 1.30)	0.97 (0.84, 1.05)	0.6
LDL: mmol/L	3.62 (2.78, 3.98)	3.55 (2.92, 3.85)	3.66 (2.73, 3.98)	0.6
Non-diabetic	46 (77%)	22 (76%)	24 (77%)	
Pre-diabetes	14 (23%)	7 (24%)	7 (23%)	

^1^ *n* (%); Median (IQR). ^2^ Calculated using one of the following tests: Fisher’s exact test (for categorical variables); Pearson’s Chi-squared test (for categorical variables, *n* > 20); Wilcoxon rank-sum exact test (for median variables, *n* < 20); Wilcoxon rank-sum test (for median variables, *n* > 20). The table presents the clinical characteristics of the study cohort, divided into two sub-cohorts based on the genotype at the study variant rs2278426. Column Description: The 1st column lists the clinical characteristics of the study cohort. The 2nd, 3rd, and 4th columns display the median value along with the interquartile range (IQR) for each clinical characteristic in the full cohort, sub-cohort with CT or TT genotype, and sub-cohort with CC genotype at rs2278426, respectively. The 5th column presents the *p* value for the difference in clinical characteristics between the sub-cohorts with the CT or TT genotype and the CC genotype. The *p* values were calculated using one of the listed statistical tests depending on the nature of the data: Fisher’s exact test in the case of dichotomous outcome; Wilcoxon rank sum test in the case of continuous outcome and when *n* < 20; Wilcoxon rank sum test in the case of continuous outcome and when *n* > 20; Pearson’s Chi-squared test in the case of categorical variables.

**Table 2:** Differences in the levels of metabolites between the individuals carrying the reference CC genotype and the individuals carrying the carrier (CT+TT) genotypes.

Metabolite	Reference (*n* = 31)	R59W (*n* = 29)	*p* Value
AC.14.1.DC.			
Mean (SD)	0.0401 (0.0129)	0.0338 (0.0114)	0.049
Median (Min, Max)	0.0410 (0.0130, 0.0660)	0.0330 (0.0130, 0.0590)	
AC.16.1.			
Mean (SD)	0.0102 (0.00380)	0.00807 (0.00393)	0.015
Median (Min, Max)	0.00900 (0.00400, 0.0230)	0.00600 (0.00300, 0.0190)	
AC.16.2.OH.			
Mean (SD)	0.00661 (0.00230)	0.00569 (0.00154)	0.014
Median (Min, Max)	0.00600 (0.00500, 0.0140)	0.00500 (0.00500, 0.0130)	
AC.18.1.			
Mean (SD)	0.0182 (0.0208)	0.0306 (0.0279)	<0.001
Median (Min, Max)	0.0110 (0.00800, 0.0830)	0.0150 (0.0100, 0.0860)	
alpha.AAA			
Mean (SD)	2.12 (0.515)	2.62 (0.714)	0.007
Median (Min, Max)	2.14 (0, 2.97)	2.45 (1.69, 4.57)	
PC.46.2.			
Mean (SD)	0.748 (0.587)	1.13 (0.954)	0.034
Median (Min, Max)	0.725 (0.00400, 2.48)	1.06 (0.108, 4.96)	
Missing	2 (6.5%)	6 (20.7%)	
PC.O.40.4.			
Mean (SD)	1.01 (0.395)	0.828 (0.306)	0.021
Median (Min, Max)	0.902 (0.372, 2.57)	0.764 (0.340, 1.72)	
Missing	0 (0%)	1 (3.4%)	
PC.O.42.4.			
Mean (SD)	0.436 (0.149)	0.369 (0.142)	0.032
Median (Min, Max)	0.389 (0.253, 0.933)	0.322 (0.172, 0.727)	
Missing	0 (0%)	1 (3.4%)	
PC.O.42.6.			
Mean (SD)	0.869 (0.243)	0.731 (0.198)	0.018
Median (Min, Max)	0.807 (0.529, 1.60)	0.711 (0.473, 1.28)	
Missing	1 (3.2%)	2 (6.9%)	
PC.O.44.6.			
Mean (SD)	1.14 (0.354)	0.924 (0.237)	0.024
Median (Min, Max)	1.06 (0.703, 2.11)	0.891 (0.504, 1.47)	
Missing	4 (12.9%)	3 (10.3%)	
CE.20.4.			
Mean (SD)	2430 (785)	2070 (678)	0.015
Median (Min, Max)	2320 (719, 4920)	1900 (1270, 4330)	
Missing	0 (0%)	1 (3.4%)	

The table presents the differences in metabolite levels between individuals carrying the reference CC genotype and individuals carrying the CT or TT genotypes at the study variant. Column Description: Metabolite column lists the specific metabolites analyzed in the study; Reference and R5W9 columns display the mean value along with the standard deviation (SD) of the metabolite levels in individuals with the CC genotype and individuals with the CT or TT genotypes; *p*-value column provides the *p*-value denoting the significance of the differences in metabolite levels associated with the statistical test used.

**Table 3:** Observed metabolites, whose levels are altered due to the R59W variant, and the effect on risk for disorders.

Metabolite	Change in Metabolite Level in the R59W Group	Disorder for which Risk Increases Due to the Observed Change in the Metabolite Level.	Reference
α-AAA	Increased	Alpha-aminoadipic acid (2-AAA) is associated with the development of type 2 diabetes (T2D) and atherosclerosis, as well as cardiometabolic disorders.	[38,59]
Phosphatidylcholine	Decreased	Masks the protective role against lysophosphatidylcholine-induced cytotoxicity and cellular senescence.	[60]
Acylcarnitine AC 18.1	Increased	Important indicators of metabolic disorders, including diabetes, cardiovascular disorders, and certain types of cancers.	[61]
Sarcosine	Decreased	It is a potential functional biomarker of the aging and diet restriction metabolic phenotype.	[42]
Cholesteryl Ester	Decreased	Accumulation of cholesterol esters in the arterial intima is a characteristic feature of atherosclerosis. This may have a protective effect due to the variant.	[62]

## Data Availability

The data presented in this study are available in the Supplementary Materials.

## Supplementary Materials

The following supporting information can be downloaded at: https://www.mdpi.com/article/10.3390/metabo13090972/s1, Table S1: Data on the levels of metabolites for each study subject along with the genotype at the study variant.
